# Ocular disease awareness and pattern of ocular manifestation in patients with biopsy-proven lung sarcoidosis

**DOI:** 10.1007/s12348-011-0029-7

**Published:** 2011-07-12

**Authors:** Maria Pefkianaki, Sofia Androudi, Anna Praidou, Vasileios Sourlas, Epameinondas Zakynthinos, Periklis Brazitikos, Konstantinos Gourgoulianis, Zoe Daniil

**Affiliations:** 1 Department of Ophthalmology, University Hospital of Larissa, Larissa, Greece; 2 Department of Ophthalmology, Aristotle University, Thessaloniki, Greece; 3 Department of Intensive Care Medicine, University of Thessaly, Larissa, Greece; 4 Department of Respiratory Medicine, University Hospital of Larissa, Larissa, Greece; 5 University of Thessalia, Mezourlo, Larissa, Greece

**Keywords:** Awareness, Ocular, Perivasculitis, Sarcoidosis, Uveitis

## Abstract

**Purpose:**

The aim of this research is to study the patterns of ocular involvement in patients with biopsy-proven lung sarcoidosis and estimate the level of patients’ awareness of possible ocular complications of sarcoidosis.

**Methods:**

Fifty patients with biopsy-proven lung sarcoidosis were referred from the Department of Respiratory Medicine, University Hospital of Larissa, Greece.

**Results:**

The most prominent ocular symptom was foreign body sensation in 15/50 patients (30%); only 6/50 of our patients (12%) were completely asymptomatic with respect to ocular symptoms. Anterior segment findings were: episcleritis in 8/50 patients (16%), iris nodules in 9/50 patients (18%), and cataract in 19/50 patients (38%). Periphlebitis was observed in 8/50 patients (16%), periarteritis in 8/50 patients (16%), epiretinal membrane in 6/50 patients (12%), and branch retinal vein occlusion in 7/50 of our patients (14%). Ten out of 50 patients (20%) had never visited an ophthalmologist before, whereas eight out of 50 patients (16%) had undergone an ophthalmic exam more than 2 years ago.

**Conclusions:**

Eye involvement is common in patient with biopsy-proven lung sarcoidosis and may occur even without prominent ocular symptoms.

## Introduction

Sarcoidosis is a multisystemic, granulomatous disease of unknown etiology that is directly characterized by non-caseating epitheloid granulomas in affected organs [1]. The disease can affect the skin, nervous system, eye, heart, liver, and a variety of other organs [2]. The etiology of the disease is unknown, although genetic, environmental, and infectious agents have been hypothesized as triggers [3]; sarcoidosis commonly affects young and middle-aged adults. On presentation, almost 90% of patients will have an abnormal chest radiograph in the form of infiltrates, not always associated with clinical symptoms or signs [4]. Ocular manifestations of the disease have been reported in up to 50% of patients with biopsy-proven disease. Two peaks of incidence were reported for ocular sarcoidosis (OS), the first at ages 20–30 years and the second at ages 50–60 years [5, 6]. Uveitis is the most common ocular manifestation (30%–70%) and is potentially vision-threatening [5–7]. The incidence of OS in industrialized countries has been found to vary, with rates reported from 27% and 20% (in London and New York, respectively) to 11% and 4% (in studies from France and Eastern Europe, respectively) [8, 9]. Even the pattern of uveitic disease has been reported to vary, with anterior uveitis being the predominant in some races (70%–75% in black ones) and posterior uveitis in others (65%–83% in white ones) [5, 6, 10]. Accurate and prompt diagnosis and treatment are important in order to preserve visual function [11].

There are limited data in the literature concerning the patterns of ocular findings in sarcoidosis and patients' awareness with respect to possible ocular involvement in sarcoidosis disease. Individual awareness is an important factor in screening, diagnosis, treatment, compliance, and prevention of possible ocular and systemic complications.

In our cohort study, patients with biopsy-proven pulmonary sarcoidosis were included and we studied the patterns of ocular involvement and estimated the patients' awareness of possible ocular complications of sarcoidosis.

## Patients and methods

Fifty patients (50) with biopsy-proven pulmonary sarcoidosis (transbronchial lung biopsy) were included in the study. All selected subjects were referred from the Department of Respiratory Medicine of the University Hospital of Larissa, Greece as part of our study protocol, which was approved by the Local Ethics Committee; a written informed consent was obtained from all study participants. This was a prospective study with the following inclusion criteria: (1) history of biopsy-proven pulmonary sarcoidosis, (2) ability to clearly communicate and understand the purpose of this study, (3) willingness to participate and ability to provide a written informed consent

In this baseline visit, we recorded demographic data, past ocular, and medical history and concomitant drug uptake. Each patient was questioned (using a standardized questionnaire) about present and past medical and ocular symptoms (possible irritation, itching, red eye, grittiness, dry eye symptoms, transient or permanent vision blurring, history of previous eye surgery) and possible ocular involvement of sarcoidosis in an effort to estimate the individual awareness of the disease ophthalmic sequelae (i.e., the patient was asked about his/hers education level, the amount of knowledge about the possible ocular sequelae of sarcoidosis, and the source of this information—health providers, textbooks, internet). All patients underwent complete ophthalmic evaluation including best-corrected visual acuity (BCVA) with Snellen charts, and slit-lamp biomicroscopy.

All patients were screened for evidence of keratoconjunctivitis sicca using Schirmer's tear test without local anesthesia. More than 15 mm moistening on the filter paper was considered as normal [12].

Intraocular pressure was recorded with a Goldman applanation tonometer. The measurement was performed using a slit lamp, with topical anesthetic and fluorescein.

We performed a non-contact fundus biomicroscopy after dilating both pupils with tropicamide and phenylephrine 5%. The posterior pole was further evaluated by slit-lamp biomicroscopy using a 90-diopter lens.

Vitreous haze was graded along an ordinal scale of 0, +0.5, +1, +2, +3, or +4 based on standard photographs developed by Nussenblatt and associates, with the modification adopted by the SUN Working Group [13, 14]. In the event of positive ocular findings, the patients were again examined at regular intervals, depending on the severity of these findings. In the event of retinal involvement, we performed fluorescein angiography within the first week of the baseline visit.

## Statistics

Data and descriptive statistics were collected prospectively for all patients. Means and standard deviations were computed for continuous variables; frequencies and percentages were calculated for categorical variables. Means, standard deviations, frequencies, and percentages were obtained. Using the chi-square test made comparisons among categorical variables for the presence of findings. Comparisons of categorical variables with continuous variable were analyzed with independent samples *t* test. Snellen visual acuity was converted to logarithm of the minimum angle of resolution units for analysis. The data were organized and analyzed using SSPS version13.0 (SSPS Inc, Chicago III). Differences giving *p* values <0.05 were defined as statistically significant.

## Results

A total of 50 Caucasians patients were enrolled, from February till June 2009. Twenty (20) of them (40%) were male and 30 (60%) were female. The mean age at presentation was 40.5 ± 10.7 years. Demographics characteristics of patients and their education level are presented in Tables 1 and 2, respectively.

**Table 1:** Demographic data of patient included in the study

	Number (%)	Age (years)
Female	30 (60%)	43 ± 10.2
Male	20 (40%)	38 ± 11.2
Total	50 (100%)	40.5 ± 10.7

**Table 2:** Patients' educational level in the study

Educational level	Patients, *n*
University/college	30
High (secondary) school	18
Primary school	2
Total	50

In ten out of 50 patients, a striking 20% had never visited an ophthalmologist before, whereas eight out of 50 patients (16%) had undergone an ophthalmic exam more than 2 years ago. The remaining 32 patients have had an ophthalmic exam within the past 2 years. The mean time elapsed from the last visit to an ophthalmologist was 19 ± 18.26 months in our study cohort. Forty-five out of 50 patients (90%) admitted that they would visit their ophthalmologist more frequently if they were diagnosed with a serious ophthalmic condition. Thirty (30) out of 50 patients (60%) were properly informed about the possible ocular complications of their systemic disease.

A direct significant correlation in favor of education level and awareness of ophthalmic complications of sarcoidosis was found (*p* < 0.05). Sex (male/female) was not directly related to the level of awareness of ocular complications of sarcoidosis (*p* = 0.518).

The most prominent ocular symptom was foreign body sensation in 15 out of 50 patients (30%); the list of ocular symptoms encountered in our study cohort is presented in Table 3. Only six out of 50 (12%) of our study patients were completely asymptomatic (with respect to ocular symptoms) at the time of their examination.

**Table 3:** Prevalence of patients' ocular sarcoidosis-related symptoms in the study

	Number of patients	Percentage (%)
None	6	12.0
Conjuctival hyperaemia	14	28.0
Itching	3	6.0
Foreign body sensation	15	30.0
Tearing	4	8.0
Floaters/veils	7	14.0
Pain	1	2.0
Blurring vision	12	24

Schirmer test was read within normal limits in 24 out of 50 patients (48%) in both eyes; abnormal readings were observed in 26 out of 50 patients (52%). Rose Bengal test was normal at the right eye in 19 patients (38%) and at the left eye in 21 patients (42%). Differences in results for both eyes, for Schirmer and Rose Bengal test, based on sex did not exist (*p* = 0.713 and *p* = 0.142 respectively).

The median BCVA for all patients was 20/20 in the better eye and 20/25 in the worse eye. The mean intraocular pressure using Goldman Applanation Tonometer was within normal limits (12.2 ± 2.5 mmHg) in all of our study subjects. Four of our patients however had glaucomatous cupping and were on intraocular pressure eye drops regularly.

Most frequent anterior segment ocular findings in our study cohort were episcleritis in eight out of 50 patients (16%), iris nodules in nine out of 50 patients (18%), and cataract in 19 out of 50 patients (38%); three out of the nine patients with iris nodules had cells in the anterior chamber. Six out of eight patients (75%) appeared with bilateral episcleritis, three out of nine patients (30%) with bilateral iris nodules and four out of 19 (20%) with bilateral cataracts, while the rest of them with unilateral appearance of each ocular finding. With respect to the cataract findings, it was not possible to clearly determine in all cases whether it was due to age, past/present steroid use, or uncontrolled inflammation.

Posterior segment findings were observed in 35 out of all patients (70%). Regarding posterior segment findings, vitreous opacities were observed in 12 out of 50 patients (24%) and in six out of 12 patients (50%) were observed bilaterally. Periphlebitis was observed in nine out of 50 patients (16%) and in four out of eight patients (50%) bilaterally, periarteritis in eight out of 50 patients (16%) and in two out of eight patients (25%) bilaterally, epiretinal membrane in six out of 50 patients (12%) and in two out of six patients (30%) bilaterally, and branch retinal vein occlusion (BRVO) in seven out of 50 patients (14%) and in three out of seven patients (40%) bilaterally. Periphlebitis was active in all of our study cases, with some degree of leaking in the fluorescein angiography. Two of the seven BRVO cases, were completely asymptomatic before; in the remaining five patients, the initial diagnosis was made prior to the enrollment in this study. The list of ocular clinical findings encountered in our study cohort is presented in Table 4.

**Table 4:** Prevalence of patients' ocular sarcoidosis-related clinical findings in the study

	Number of patients	Percentage (%)
Extraocular		
Episcleritis	8	16
Intraocular		
Iris nodules	9	18
Retinal perivasculitis (periphlebitis, periarteritis)	16	32
Cataract	19	38
Vitreal opacities (vitreous haze)	12	24
Epiretinal membrane	6	12
Branch retinal vein occlusion	7	14

We did not find any direct correlation between sex and present ocular symptoms (*p* = 0.340), but we observed sex-specific differences in cataract prevalence between males and females. Fourteen out of 30 female patients (46.7%), as opposed to only four out of 20 male patients (20%) presented with cataract, and this result was statistically significant (*p* = 0.022).

We also noted a statistical significant difference in the occurrence of retinal involvement and the duration of sarcoidosis: Thirty-seven out of 50 patients (74%) with lung disease more than 48 months were more likely to have periphlebitis, periarteritis, epiretinal membrane, or neovascularization as compared to 12 out of 50 patients (26%) with lung disease duration of less than 48 months; this result was statistically significant (*p* = 0.032).

## Discussion

Sarcoidosis is a multisystem granulomatous disorder. Ocular manifestations are common in 25–50% of patients with histologically proven disease [15]. Although ocular sarcoidosis more commonly presents as an anterior granulomatous uveitis, a wide spectrum of ocular involvement exists. Typical posterior segment findings of sarcoidosis include vitritis with or without inflammatory “snowballs”, retinal periphlebitis, preretinal inflammatory nodules, and “candle wax drippings” [10, 16]. Choroidal lesions have also been reported in ocular sarcoidosis [16, 17].

Guidelines for diagnosis of ocular lesions in sarcoidosis are the followings: (1) mutton-fat keratic precipitates and/or iris nodules (Koeppe/Busacca), (2) trabecular meshwork nodules and/or tent-shaped peripheral anterior synechia, (3) vitreous opacities (snowball/string of pearls-like appearance), (4) multiple chorioretinal peripheral lesions, (5) retinal perivasculitis (periphlebitis) and/or candlewax retinochoroidal exudates and/or laser photocoagulation spots-like retinochoroidal atrophy, (6) optic disc nodule(s)/granuloma(s) and/or solitary choroidal nodule, and (7) bilaterality [18, 19]. Cases meeting two or more of the above categories fall into the clinically suspected sarcoidosis group, entitled to further testing for histologic confirmation.

In this study, we present the wide spectrum of clinical presentation of ocular sarcoidosis (Tables 3 and 4). In addition, we assess and estimate the level of awareness of ocular involvement, among patients with histological proven pulmonary sarcoidosis.

Our results demonstrate a direct significant correlation in favor of education level and awareness of ophthalmic complications of sarcoidosis. There is no safe conclusion with respect to whether this awareness stems from individual perception ability, the fact that an educated individual has more easily access to various informational sources (internet, interaction with medical specialists), or the quality of information from primary care physicians. The fact that all of our study patients came from the same original source (Department of Respiratory Medicine of our Institution) favors the individual ability and perception as the strongest factor.

We also observed statistically significant sex-specific differences in cataract prevalence between males and females. Because of inherent difficulties in grading lens opacities in this study, we do not include tabulations of cataract formation. It is likely that the higher prevalence of cataract in our female study population can be partially attributed to their older age, but even after adjusting for the age difference, the sex difference in cataract formation still exists. We also noted a statistical significant difference in the occurrence of retinal involvement and the duration of sarcoidosis: patients with lung disease of more than 48 months were more likely to have periphlebitis, periarteritis, epiretinal membrane, or neovascularization compared to patients with lung disease duration of less than 48 months.

The differential diagnosis of ocular sarcoidosis is variable according to the specific clinical manifestations and can mimic almost any other disease, including anterior, intermediate, or posterior uveitis, panuveitis, or a masquerade syndrome. Our study results demonstrate a positive correlation between patients' education and level of awareness for potentially vision-robbing sequelae of sarcoidosis. Of course, this finding may be somewhat biased by the fact that some of our cases had already experienced a severe ocular involvement (i.e., BRVO) and they might have sought some additional resources for sarcoidosis disease with respect to its ocular involvement. Despite this limitation, and taking into account the fact of positive correlation of educational level and disease awareness, our study results demonstrate that hospital social services should focus on the correct and prompt information of ocular sequelae of systemic diseases, for patient that are educationally deprived.

## References

[1] Newman LS, Rose CS, Maier LA (1997). Sarcoidosis. N Engl J Med.

[2] Baughman RP, Lower EE, du Bois R (2003). Sarcoidosis. Lancet.

[3] Moller DR (1997). Aetiology of sarcoidosis. Clin Chest Med.

[4] Neville E, Walker AN, James DG (1983). Prognostic factors predicting the outcome of sarcoidosis: an analysis of 818 patients. Q J Med.

[5] Rothova A (2000). Ocular involvement in sarcoidosis. Br J Ophthalmol.

[6] Rothova A, Alberts C, Glasius E (1989). Risk factors for ocular sarcoidosis. Doc Ophthalmol.

[7] Bradley D, Baughman RP, Raymond L, Kaufman AH (2002). Ocular manifestations of sarcoidosis. Semin Respir Crit Care Med.

[8] James DG (1986). Ocular sarcoidosis. Ann N Y Acad Sci.

[9] James DG, Hosoda Y, James DG (1994). Epidemiology. Sarcoidosis and other Granulomatous Diseases.

[10] Hunter DG, Foster CS, Albert DM, Jakobiec FA (1994). Ocular manifestations of sarcoidosis. Principles and Practice of Ophthalmology.

[11] Krzystolik M, Power WJ, Foster CS (1998). Diagnostic and therapeutic challenges of sarcoidosis. Int Ophthalmol Clin.

[12] Lemp MA (1995). Report of the National Eye Institute/Industry workshop on clinical trials in dry eyes. CLAO J.

[13] Jabs DA, Nussenblatt RB, Rosenbaum JT (2005). Standardization of uveitis nomenclature for reporting clinical data. Results of the First International Workshop. Am J Ophthalmol.

[14] Nussenblatt RB, Palestine AG, Chan CC, Roberge F (1985). Standardization of vitreal inflammatory activity in intermediate and posterior uveitis. Ophthalmology.

[15] Khanna A, Sidhu U, Bajwa G, Malhotra V (2007). Pattern of ocular manifestations in patients with sarcoidosis in developing countries. Acta Ophthalmol Scand.

[16] Smith JA, Foster CS (1996). Sarcoidosis and its ocular manifestations. Int Ophthalmol Clin.

[17] Ghabrial R, McCluskey PJ, Wakefield D (1997). Spectrum of sarcoidosis involving the eye and brain. Aust N Z J Ophthalmol.

[18] Asukata Y, Ishihara M, Hasumi Y (2008). Guidelines for the diagnosis of ocular sarcoidosis. Ocul Immunol Inflamm.

[19] Herbort CP, Rao NA, Mochizuki M, members of Scientific Committee of First International Workshop on Ocular Sarcoidosis (2009). International criteria for the diagnosis of ocular sarcoidosis: results of the first International Workshop on Ocular Sarcoidosis (IWOS). Ocul Immunol Inflamm.

